# The value of DCE- MRI of the breast as a diagnostic tool in assessing amorphous calcifications in screening mammography

**DOI:** 10.3389/fonc.2023.1151500

**Published:** 2023-04-25

**Authors:** Siqi Wang, Hui Wang, Yang Li, Jianjuan Lou, Qigui Zou, Yanni Jiang, Feiyun Wu, Yuxia Tang, Shouju Wang

**Affiliations:** ^1^ Department of Radiology, the First Affiliated Hospital of Nanjing Medical University, Nanjing, China; ^2^ Department of Radiology, Nanjing Hospital of Chinese Medicine Affiliated to Nanjing University of Chinese Medicine, Nanjing, China

**Keywords:** breast cancer, mammography, magnetic resonance imaging, microcalcifications, biopsy

## Abstract

**Purpose:**

To evaluate the diagnostic performance of dynamic contrast-enhanced magnetic resonance imaging in differentiating benign and malignant amorphous calcifications.

**Methods:**

This study included 193 female patients with 197 suspicious amorphous calcifications detected on screening mammography. The patients’ demographics, clinical follow-up, imaging, and pathology outcomes were reviewed, and sensitivity, specificity, positive predictive value (PPV), and negative predictive value (NPV) of DCE-MRI were calculated.

**Results:**

Of 197 lesions (193 patients) included in the study, 50 (25.4%) were histologically proved to be malignant. DCE-MRI based on breast imaging report and diagnosis system (BI-RADS) had a sensitivity of 94.4%, specificity of 85.7%, PPV of 69.1%, and NPV of 97.7% for the detection of malignant amorphous calcifications. Notably, diagnosis solely based on the presence or absence of DCE-MRI enhancement showed the same sensitivity but significantly decreased specificity (44.8%, p < 0.001) and PPV (44.8%, p < 0.001). In patients with a minimal or mild degree of background parenchymal enhancement (BPE), the sensitivity, specificity, PPV, and NPV increased to 100%, 90.6%, 78.6%, and 100%, respectively. However, in patients with a moderate degree of BPE, MRI resulted in three false negatives of ductal carcinoma *in situ* (DCIS). Overall, the addition of DCE-MRI detected all invasive lesions and could decrease unnecessary biopsy by 65.5%.

**Conclusion:**

DCE-MRI based on BI-RADS has the potential to improve the diagnosis of suspicious amorphous calcifications and avoid unnecessary biopsy, especially for those with low-degree BPE.

## Introduction

Dynamic contrast-enhanced magnetic resonance imaging (DCE-MRI) is a highly sensitive imaging modality for breast cancer detection, with reported sensitivity up to 100% in patients with suspicious lesions (1–3).However, the diagnostic value of DCE-MRI for suspicious microcalcifications detected by mammography is a topic of debate (4–9),and the current guideline does not recommend downgrading suspicious calcifications based on benign MRI appearance. Recent meta-analyses suggest that the absence of enhancement on DCE-MRI can be used to rule out malignancy for some BI-RADS 4 microcalcifications and avoid unnecessary biopsy (10, 11). However, the value of DCE-MRI for microcalcifications with certain morphologic descriptors, particularly amorphous calcifications, remains unclear.

Amorphous calcifications are small microcalcifications that appear so hazy that no specific morphologic descriptors can be assigned. The management of amorphous calcifications has varied from surveillance to biopsy, and the reported malignancy rate of amorphous calcification varies widely (12–15). Several studies have raised the question of whether amorphous calcifications can be further stratified to decrease the number of benign biopsies (16).

Therefore, the purpose of this study was to evaluate the diagnostic value, particularly the negative predictive value, of DCE-MRI in differentiating between benign and malignant amorphous calcifications and to determine if it could be used to avoid unnecessary biopsy.

## Materials and methods

### Study subjects

The institutional review board of our hospital approved this study. Given its retrospective design, written informed consent was waived.

We performed a retrospective search of biopsies carried out between December 1, 2016, and March 31, 2022, on microcalcifications deemed suspicious by radiologists on screening mammography. We included cases that (1) underwent breast MRI before biopsy; (2) had amorphous microcalcification morphology. We excluded cases that (1) had microcalcifications associated with mass, distortion, or asymmetry; (2) had an interval of more than three months between mammography and DCE-MRI.

### Mammography protocol and interpretations

In our department, we performed bilateral digital diagnostic mammography in standard projections, including craniocaudal and mediolateral oblique views, using the unit (Selenia, Hologic) under auto-filter mode.

### MRI protocol and interpretations

As per our institutional workflow, we recommended MRI to patients with suspicious microcalcifications to further assess the microcalcifications before biopsy. All patients underwent MR imaging in the prone position using 1.5 or 3 Tesla units (MGAGNETOM Aera XJ, Siemens and MAGNETOM Trio, Siemens, respectively). The protocol included at least a localization sequence, axial Tirm (turbo inversion recovery magnitude) sequence, Diffusion Weighted Imaging (DWI), DCE sequence with fat suppression, and sagittal fat-suppressed T2 weighted imaging (T2WI). Gadolinium-DTPA (Magnevist; Bayer Healthcare) was injected at a dose of 0.1 mmol/kg and a rate of 3 mL/s, followed by a 20 mL saline solution.

The imaging protocol of MAGNETOM Trio was as follows: (1) axial Tirm (TR/TE,3450/61 ms; field of view, 340 mm × 340 mm; matrix, 320 × 320; flip angle, 80˚; slice thickness, 4 mm); (2) axial DWI (b value of 50/800 s/mm^2^; TR/TE, 5200/65 ms; field of view, 323 mm × 161 mm; matrix, 220 × 110; flip angle, 180˚; slice thickness, 5 mm); (3) DCE sequence (TR/TE, 4.23/1.57ms; field of view, 340 mm × 340 mm; matrix, 448 × 448; slice thickness, 1 mm; flip angle, 10˚; pixel resolution, 0.8 × 0.8 × 1.0 mm^3^; temporal resolution, 1 min) was acquired before and repeated five times after contrast agent administration; (4) sagittal fat-suppressed T2WI (TR/TE, 2910/72 ms; field of view, 200 mm × 200 mm; matrix, 320× 320; flip angle, 80˚; slice thickness, 4 mm). The total scan time was 16 min 55 sec.

The imaging protocol of MAGNETOM Aera was as follows: (1) axial Tirm (TR/TE, 5320/57 ms; field of view, 340 mm × 340 mm; matrix, 384×384; flip angle, 150˚; slice thickness, 4 mm); (2) axial DWI (b value of 50/800 s/mm^2^; TR/TE, 7500/64 ms; field of view, 350 mm × 163 mm; matrix, 180 × 84; flip angle, 180˚; slice thickness, 5 mm); (3) DCE sequence (TR/TE, 3.90/1.66 ms; field of view, 360 mm × 360 mm; matrix, 320 × 320; flip angle, 10˚; slice thickness, 1.5 mm; pixel resolution, 1.1 × 1.1 × 1.5 mm^3^; temporal resolution, 1 min) was acquired before and repeated five times after contrast agent administration; (4) sagittal fat-suppressed T2WI (TR/TE, 4000/70 ms; field of view, 250 mm × 250 mm; matrix, 320 × 320; flip angle, 150˚; slice thickness, 4 mm). The total scan time was 18 min 6 sec.

### Image interpretations and management

The acquired images were independently interpreted by two expert radiologists, each with over 10 years of experience in breast imaging. The radiologists were blinded to the pathological results to ensure unbiased interpretation. The corresponding area of suspicious amorphous calcification on MRI was analyzed, and the diagnosis was made based on BI-RADS or the presence of enhancement. For BI-RADS-based diagnosis, breast MRI was evaluated using the 5th BI-RADS lexicon with knowledge of the mammographic findings. Patients diagnosed as BI-RADS 4 and 5 were considered positive, while those diagnosed as BI-RADS 1, 2, and 3 were considered negative. For diagnosis based on enhancement presence, patients were considered positive if there was any enhancement which appeared in the area corresponding to the calcifications. In case of controversial findings, a consensus diagnosis was made by the two radiologists. All patients with suspicious amorphous calcifications were recommended for biopsy.

### Statistical analysis

The diagnostic performance of DCE-MRI was calculated in terms of sensitivity, specificity, positive predictive value (PPV), and negative predictive value (NPV). The performance comparison between BI-RADS-based diagnosis and diagnosis based on the presence of enhancement was evaluated using McNemar’s test. The performance comparison between DCE-MRI performed on 1.5 and 3 Tesla scanners was evaluated using Fisher’s test. Demographic data comparison between patients diagnosed as benign and malignant was performed using t-test or Fisher’s test. A p-value of less than.05 was considered significant. All analyses were performed using R software packages.

## Results

### Patient cohort

**Figure 1:**
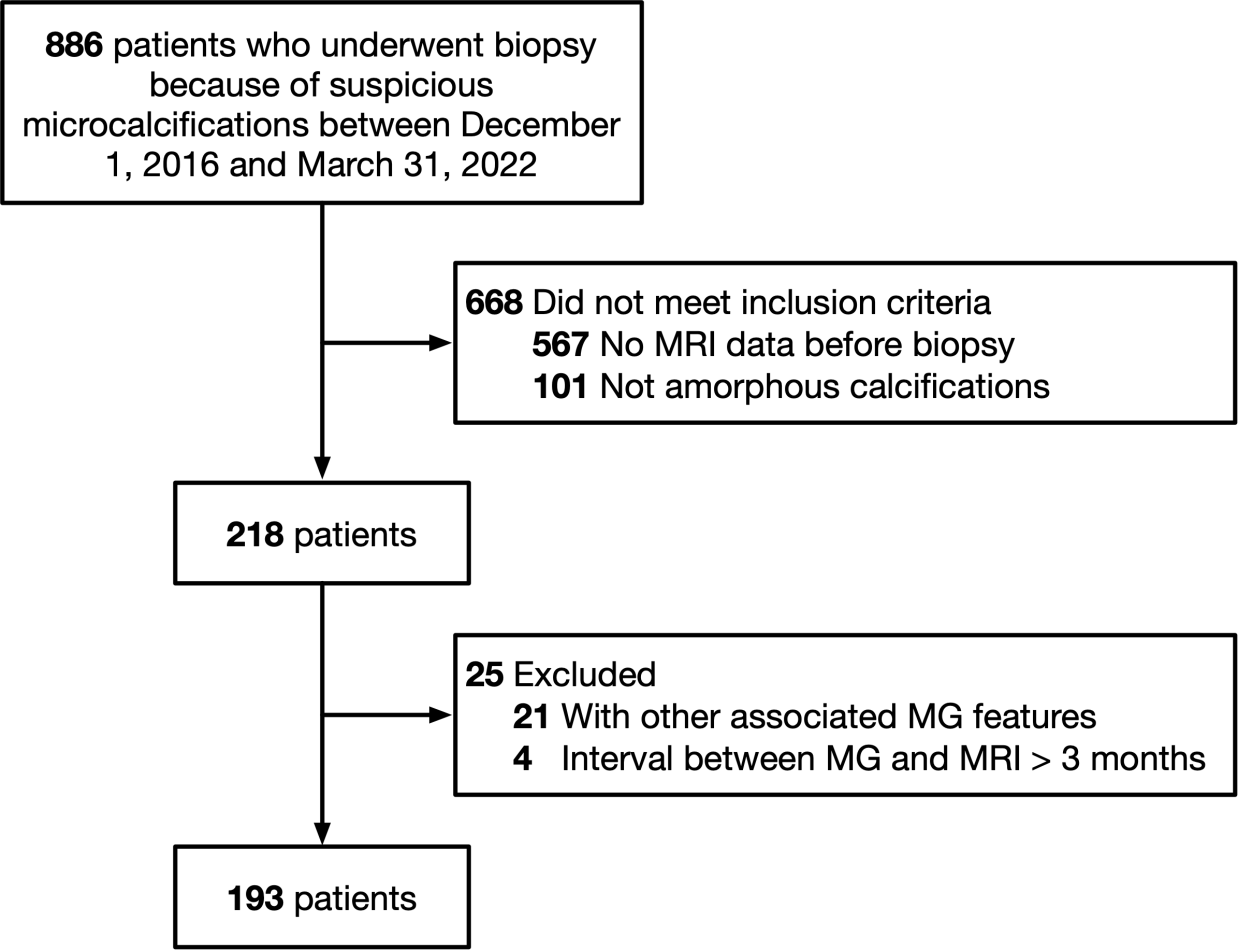
Flowchart of subject inclusion and exclusion criteria.

Between December 1, 2016, and March 31, 2022, a total of 886 patients with suspicious mammography findings underwent biopsy. Among these, 668 patients were excluded because they did not meet the inclusion criteria. Of the remaining 218 patients, 21 were excluded because the amorphous calcifications were associated with other features such as mass, distortion, or asymmetry. Additionally, four cases were excluded because DCE-MRI was performed three months after the initial mammography (**Figure 1**). The final dataset included 193 female patients with a median age of 46.5 years (age range, 24-68 years). The median interval between mammography and DCE-MRI was five days (range, 0-69 days). Of the 193 patients, eight underwent Stereotactic Vacuum-Assisted Biopsy (SVAB), and the other 185 underwent surgical biopsy. Fifty (25.9%) patients were histologically proven to have malignancy. The demographic details of the patients are listed in **Table 1**.

**Table 1:** Demographic data (n = 193).

	Total (n = 193)	Benign (n = 143)	Malignant (n = 50)	P value^1^
Age, y	46.2 ± 8.3	45.4 ± 7.7	48.7 ± 9.5	0.02
Menopausal status				
Premenopausal	143 (74.1%)	110 (76.9%)	33 (66%)	0.13
Postmenopausal	50 (25.9%)	33 (23.1%)	17 (34.0%)	
Family history of breast cancer				
Yes	11 (5.7%)	9 (6.3%)	2 (4.0%)	0.73
No	182 (94.3%)	134 (93.7%)	48 (96.0%)	
Personal history of breast cancer				
Yes	6 (3.1%)	1 (0.7%)	5 (10.0%)	0.004
No	187 (96.9%)	142 (99.3%)	45 (90.0%)	

^1^Comparison between patients diagnosed as benign and malignant.

There were 197 microcalcifications detected in the cohort of 193 female patients. Of these 197 lesions, 50 (25.4%) were found to have histologically proven malignancy, including 30 ductal carcinomas *in situ* (DCIS), 1 invasive ductal carcinoma (IDC), 15 IDC associated with DCIS, 2 invasive lobular carcinomas, and 2 solid papillary carcinomas. The remaining 147 (74.6%) cases were benign, consisting of 131 fibrocystic changes, 8 fibroepithelial tumors, 1 lobular carcinoma *in situ* (LCIS), 2 atypical hyperplasia, and 5 other benign lesions.

### Mammography

Regarding the mammography findings, the 197 lesions detected were distributed as follows: 49 (24.8%) lesions had a regional distribution, 124 (62.9%) lesions had a grouped distribution, 4 (2.0%) lesions had a linear distribution, and 20 (10.2%) lesions had a ductal distribution. The malignancy rates for suspicious amorphous calcifications were 12.2% (6 of 49), 25.0% (31 of 124), 75% (3 of 4), and 50% (10 of 20) for the regional, grouped, linear, and ductal distribution, respectively. Overall, the positive predictive value (PPV) of suspicious amorphous calcifications on mammography was 25.4%.

### DCE-MRI

The DCE-MRI was performed using a 1.5 (64 cases, 33.2%) or 3 (129 cases, 66.8%) Tesla scanner. Among the 197 lesions, 92 (46.7%) showed no enhancement in the corresponding area on DCE-MRI. The remaining 105 (53.3%) lesions were further classified into 30 focus enhancement, 9 mass enhancement, and 66 non-mass enhancement. The diagnosis and characteristics of DCE-MRI were tabulated in **Table 2**.

**Table 2:** Diagnosis and characteristics of DCE-MRI for suspicious amorphous calcifications.

	Total (n = 197)	Benign (n =147)	Malignant (n = 50)
BI-RADS			
1	92 (46.7%)	89 (60.5%)	3 (6.0%)
2	23 (11.7%)	23 (15.6%)	0 (0%)
3	14 (7.1%)	14 (9.5%)	0 (0%)
4	68 (34.5%)	21 (14.3%)	47 (94.0%)
Enhancement			
No	92 (46.7%)	89 (60.5%)	3 (6.0%)
Yes	105 (53.3%)	58 (39.5%)	47 (94.0%)
Focus	30 (28.6%)	28 (48.3%)	2 (4.3%)
Mass	9 (8.6%)	3 (5.2%)	6 (12.8%)
NME	66 (62.9%)	27(46.6%)	39 (83.0%)
Background parenchymal enhancement			
Minimal	71 (36.0%)	49 (33.3%)	22 (44.0%)
Mild	72 (36.5%)	57 (38.8%)	15 (30.0%)
Moderate	43 (21.8%)	32 (21.8%)	11 (22.0%)
Marked	11 (5.6%)	9 (6.1%)	2 (4.0%)

Out of the 197 lesions, 105 (53.3%) had DCE-MRI findings correlating with the location of suspicious amorphous calcifications, among which 47 were malignant and 58 were benign. The remaining 92 lesions (46.7%) that showed no findings on DCE-MRI were benign in 89 cases and malignant in 3 cases. The degree of background parenchymal enhancement (BPE) was also documented, with 143 lesions having low-degree BPE (minimal or mild) and 54 lesions having high-degree BPE (moderate or marked). There was no significant difference in BPE degree between benign and malignant lesions (low-degree: 106 vs. 37; high-degree: 41 vs. 13; p = 0.85).

The lesions were classified according to BI-RADS lexicons, with 129 (65.5%) lesions classified as negative (BI-RADS 1-3) and 68 (34.5%) lesions classified as positive (BI-RADS 4). None of them were classified as BI-RADS 5. The subgroup analysis of MRI BI-RADS classification was presented in **Table 3**.

**Table 3:** Diagnosis performance of DCE-MRI for suspicious amorphous calcifications.

	Sensitivity	Specificity	PPV	NPV
Criterion^1^				
BI-RADS	94.0% (p = 1.00)	85.7% (p < 0.01)	69.1% (p < 0.01)	97.7% (p = 0.09)
Enhancement	94.0%	60.5%	44.8%	96.7%
Field Strength^2^				
1.5 T	86.7% (p = 0.21)	82.4% (p = 0.46)	59.1% (p = 0.27)	95.9% (p = 0.27)
3 T	97.1%	87.5%	73.9%	98.8%
BPE degree^2^				
Low	100% (p = 0.01)	90.6% (p = 0.01)	78.7% (p = 0.02)	100% (p = 0.01)
High	76.9%	73.2%	47.6%	90.9%

^1^McNemar’s test was performed for comparison.

^2^ Fisher’s test was performed for comparison. Low-degree BPE includes mild and moderate degrees of BPE. High-degree BPE includes moderate and marked degrees of BPE.

**Figure 2:**
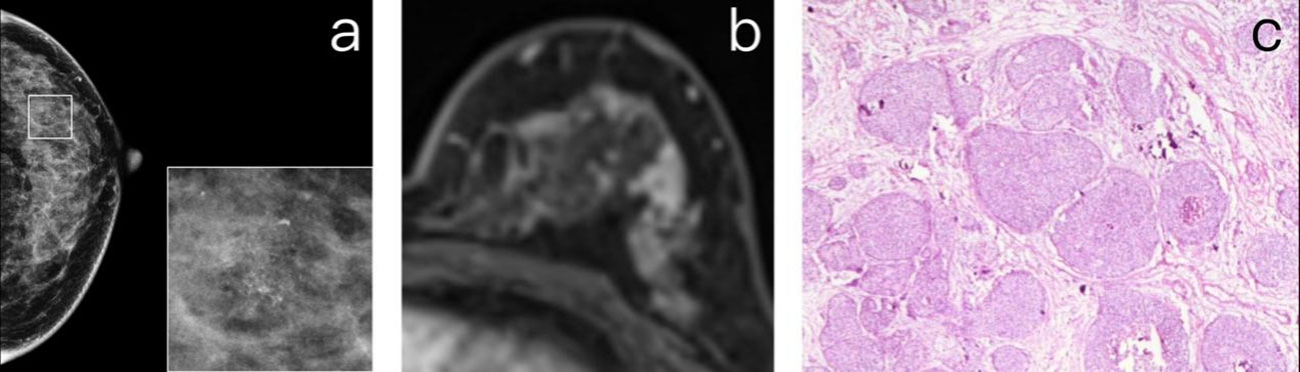
**(A)** A 47-year-old premenopausal female who underwent screening mammography shows grouped amorphous microcalcifications in the upper outer quadrant of the left breast. **(B)** Postcontrast DCE-MR images of the left breast show regional NME correlated to the location of calcifications. The patient was diagnosed as BI-RADS 4. **(C)** The patient underwent breast-conserving surgery. The histopathology shows a grade 2 DCIS.

The sensitivity, specificity, PPV, and NPV of DCE-MRI for suspicious amorphous calcification based on BI-RADS classification were 94.0%, 85.7%, 69.1%, and 97.7%, respectively (**Table 4**). When using enhancement presence instead of BI-RADS lexicon to diagnose the amorphous calcification, the sensitivity remained at 94.0%, but the specificity, PPV, and NPV dropped to 60.5%, 44.8%, and 96.7%, respectively. There was no significant difference in diagnostic performance between 1.5 and 3 Tesla MRI in terms of sensitivity, specificity, PPV, and NPV. However, DCE-MRI showed significantly higher performance for lesions with low-degree BPE than those with high-degree BPE. Of note, in patients with low-degree BPE, all malignant lesions were detected with no false negatives (**Figure 2**).

**Table 4:** Breakdown of MRI BI-RADS diagnosis and pathological results according to the distribution of microcalcification on mammography.

Distribution	MRI BI-RADS	Benign	Malignant	Sum
Regional	Negative	36 (100%)	0	36
	1	24 (100%)	0	24
	2	7 (100%)	0	7
	3	5 (100%)	0	5
	Positive			
	4	7 (53.8%)	6 (46.2%)	13
Grouped	Negative	82 (96.5%)	3 (3.5%)	85
	1	63 (95.5%)	3 (0.5%)	66
	2	13 (100%)	0	13
	3	6 (100%)	0	6
	Positive			
	4	11 (28.2%)	28 (71.8%)	39
Linear	Negative	0	0	0
	Positive	1 (25.0%)	3 (75.0%)	4
Ductal	Negative	8 (100%)	0	8
	1	2 (100%)	0	2
	2	3 (100%)	0	3
	3	3 (100%)	0	3
	Positive			
	4	2 (16.7%)	10 (83.3%)	12

### False negatives and positives at DCE-MRI

**Figure 3:**
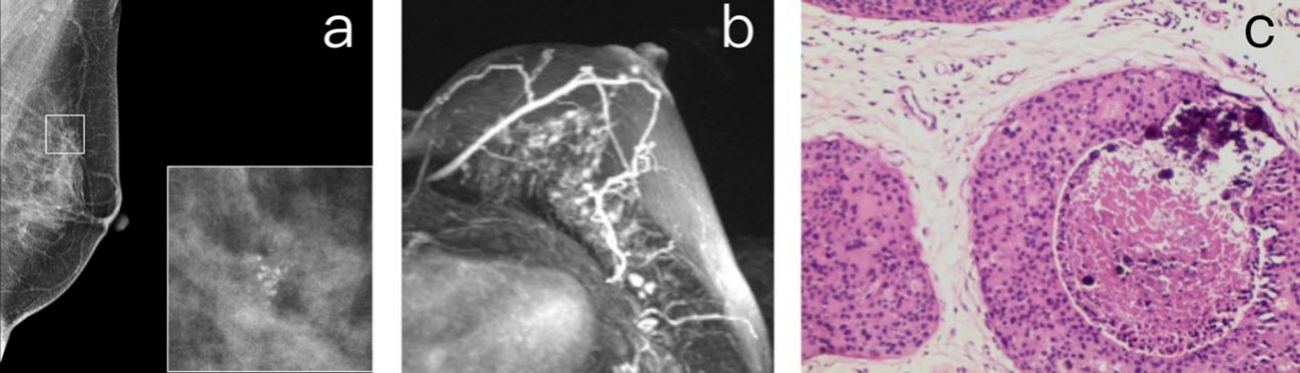
**(A)** A 55-year-old premenopausal female who underwent screening mammography shows grouped amorphous microcalcifications in the upper quadrant of the left breast. **(B)** Maximum intensity projection reconstructed from subtracted images of DCE-MR study. The images show a moderate degree of BPE in the left breast but no suspicious enhancement in the correlated location of calcifications. The patient was diagnosed as BI-RADS 1. **(C)** The histopathology shows a grade 2 DCIS.

All three false negatives observed in DCE-MRI were cases of intermediate-grade DCIS, as detailed in **Table 5**. The median size of the lesion was 5.0 mm, and all three patients had a moderate degree of background parenchymal enhancement (BPE) on DCE-MRI (**Figure 3**).

**Table 5:** Characteristics of false negatives at DCE-MRI.

Case	Age, y	Menopausal status	Family history of breast cancer	Personal history of breast cancer	Type of cancer	Largest diameter, mm	Maximum reported grade	ER status	PR status	HER2 status	BPE degree
1	45	Premenopausal	No	No	DCIS	5	2	Positive	Positive	Negative	Moderate
2	55	Premenopausal	No	No	DCIS	7	2	Positive	Positive	Negative	Moderate
3	44	Premenopausal	No	No	DCIS	2	2	Positive	N.A.	N.A.	Moderate

ER, estrogen receptor; PR, progesterone receptor; HER2, human epidermal growth factor receptor 2.

**Figure 4:**
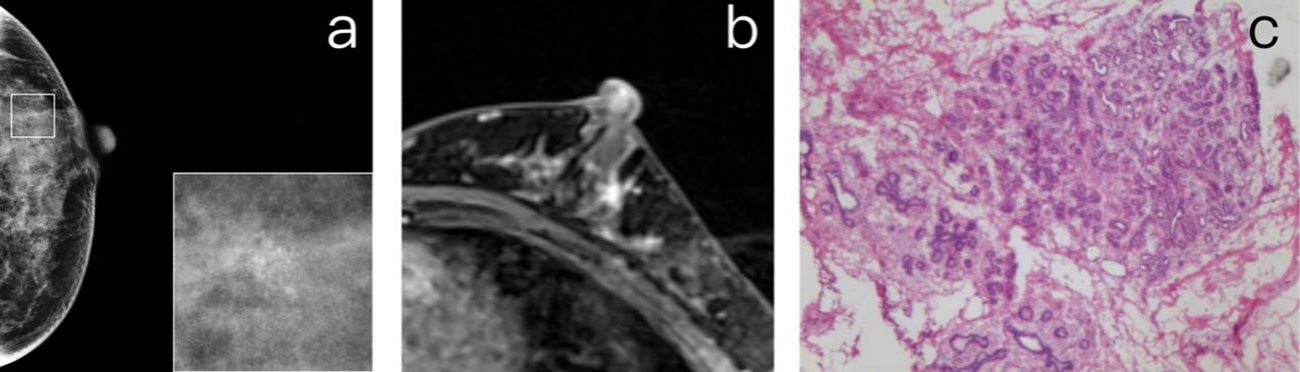
**(A)** A 40-year-old premenopausal female who underwent screening mammography shows grouped amorphous microcalcifications in the upper outer quadrant of the left breast. **(B)** Postcontrast DCE-MR images of the left breast show focal NME correlated to the location of calcifications. The patient was diagnosed as BI-RADS 4. **(C)** The histopathology shows fibrocystic changes, including components of sclerosing adenosis, columnar cell change, microcalcifications, and focal usual ductal hyperplasia.

Among the 21 false positives diagnosed using the BI-RADS criteria, all but one were non-mass enhancement (NME). The distribution of NME included 9 focal, 2 ductal, 1 segmental, and 8 regional distributions. Of the 21 false positives, 16 were histologically identified as fibrocystic change, 1 was a fibroepithelial tumor, 1 was LCIS, 1 was atypical hyperplasia, and 1 was an intraductal papilloma (**Figure 4**).

None of the 193 patients showed incidental MRI positive findings classified as BI-RADS 4 or 5.

## Discussion

In this study, we aimed to investigate the diagnostic performance of DCE-MRI at suspicious amorphous calcifications retrospectively. Based on BI-RADS, the sensitivity and NPV of DCE-MRI were found to be 94.0% and 97.7%, respectively. For patients with low-degree BPE, the sensitivity and NPV increased to 100%. Overall, the addition of DCE-MRI after mammography could decrease 65.5% (129/197) of the biopsy, and the PPV increased from 25.4% to 69.1%, with 3 false negatives of non-high-grade DCIS. Although the diagnostic value of DCE-MRI in mammographic microcalcifications has been investigated in several reports, to the best of our knowledge, no study has specifically analyzed that in the case of suspicious amorphous calcifications (1–3, 5–7, 17–26). We included the largest dataset (n = 197) of suspicious amorphous calcifications to date. Our results indicate that DCE-MRI is a powerful tool to stratify the risk of amorphous calcifications, with the NPV of DCE-MRI based on BI-RADS reaching 97.7%.

A meta-analysis of 20 studies suggested using the presence of enhancement at the area of microcalcification as the diagnostic criterion of added DCE-MRI (11). However, our results showed that the diagnosis based on the MRI BI-RADS classification has the same sensitivity but higher specificity, PPV, and NPV than that based on the presence of enhancement. These results are consistent with those reported by Ninno et al. (specificity increased from 77.8% to 86% with unchanged sensitivity) and Luo et al. (specificity increased from 50.5% to 58.1% with similar sensitivity) (6, 26).

The subgroup analysis revealed that the performance of DCE-MRI was influenced by the degree of background parenchymal enhancement (BPE), but not by the magnetic strength of scanners. For patients with low-degree BPE, the sensitivity and negative predictive value (NPV) of DCE-MRI achieved 100%. However, for those with high-degree BPE, the sensitivity and NPV dropped to 73.2% and 90.9%. Therefore, caution is warranted when downgrading amorphous calcifications in these patients, as the lesions may be obscured in areas with moderate or marked BPE. Furthermore, the specificity (73.2% vs 90.6%) and positive predictive value (PPV) (47.6% vs 78.7%) of DCE-MRI were also lower in patients with high-degree BPE than those with low-degree BPE. This is because BPE is difficult to distinguish from non-mass enhancement (NME) when it manifests with a focal or regional distribution and increases the possibility of being interpreted as malignant by radiologists (27), which is consistent with our finding that most of the false positives (20 out of 21) were NME. Future studies investigating the variation in patterns and degrees of BPE may help improve the diagnostic accuracy of DCE-MRI in diagnosing suspicious amorphous calcifications.

Our study found that DCE-MRI was able to detect all invasive lesions and 90% (27 of 30) of DCIS. However, the three false negatives of DCE-MRI were non-high-grade DCIS. In a similar study by Di Ninno et al, eight false negatives were reported for DCE-MRI detection of microcalcifications, including six non-high-grade DCIS and two high-grade DCIS. This suggests that low-to-medium grade DCIS may be undetectable by DCE-MRI due to their small size and low-level neovascularization (26). Non-high-grade DCIS also tend to have more biologically indolent behavior, and a recent study found no survival benefit of surgery for patients with low-grade DCIS (28). Therefore, active surveillance may still be an option for those suspicious amorphous microcalcifications with negative DCE-MRI findings in the future, even if they are non-high-grade DCIS.

Several limitations of our study should be noted. Firstly, it was a retrospective study conducted at a single institution, which might limit the generalizability of the results to other populations. Secondly, we did not analyze the effect of the size of microcalcification clusters on the performance of MRI. However, accurately determining the size of amorphous microcalcifications can be challenging due to their indistinct appearance. Lastly, there could be interobserver variability in the interpretation of MRI findings. Although we did not evaluate the variability between the two radiologists, the final diagnosis was made by their consensus, which reflects the routine practice of our institution.

## Conclusions

In conclusion, our study suggests that DCE-MRI has the potential to improve the diagnosis of suspicious amorphous calcifications, regardless of the magnetic strength of the scanner used. The BI-RADS classification-based criterion had a similar sensitivity to that based on the presence of enhancement, but it significantly improved the specificity of the diagnosis. DCE-MRI demonstrated better performance in patients with minimal or mild background parenchymal enhancement. The addition of DCE-MRI increased the positive predictive value of amorphous calcifications, which may lead to a reduction in unnecessary biopsies.

## Data availability statement

The datasets presented in this article are not readily available because of protection of patient privacy. The datasets are accessible with reasonable request from the corresponding author. Requests to access the datasets should be directed to Wang S, shouju.wang@gmail.com.

## Ethics statement

The studies involving human participants were reviewed and approved by Ethics Committee of the First Affiliated Hospital of Nanjing Medical University. Written informed consent for participation was not required for this study in accordance with the national legislation and the institutional requirements.

## Author contributions

YT and SJW: designed the study. SQW, HW, JL, QZ, and YJ: collected the data. YL and YT: analyzed the data. SQW, HW, and SJW: prepared the manuscript. FW: supervised the study. All authors read and approved the final manuscript.
